# Barriers to healthcare access for irregular immigrants after their arrival in Spain: a systematic review

**DOI:** 10.1093/eurpub/ckaf042

**Published:** 2025-04-09

**Authors:** Javier Fagundo-Rivera, María Soledad García-Lozano, Francisco Javier Portero-Prados, Rocío Romero-Castillo, Nadine Badillo-Sánchez, Pablo Fernández-León

**Affiliations:** Centro Universitario de Enfermería Cruz Roja, University of Seville, Seville, Spain; Infanta Leonor Hospital, Madrid Health Service, Madrid, Spain; Centro Universitario de Enfermería Cruz Roja, University of Seville, Seville, Spain; Intensive Care Area, Virgen del Rocío University Hospital, Andalusian Health Service, Junta de Andalucia, Seville, Spain; Centro Universitario de Enfermería Cruz Roja, University of Seville, Seville, Spain; Department of Nursing, University of Seville, Seville, Spain; School of Doctorate, University of Huelva, Huelva, Spain; Centro Universitario de Enfermería Cruz Roja, University of Seville, Seville, Spain; School of Doctorate, University of Seville, Seville, Spain

## Abstract

Examining the barriers encountered by irregular immigrants in accessing the public health system is crucial for the continuity of healthcare processes. This approach not only heightens patient-centered care but also fosters long-term public health preparedness and social cohesion. The aim of this review was to examine the existing barriers to accessing the Spanish healthcare system for the immigrant population. A systematic review of original articles was conducted based on the PRISMA methodology. Studies registered in PubMed, Scopus, CINAHL, LILACS, Web of Science, and Enfispo were analyzed. A total of 4773 articles were identified, of which 15 were selected for review. Among the selected articles, 10 employed qualitative methodologies, 1 utilized a mixed methodology, and 4 used quantitative methodologies. A variety of access barriers related to communication, administrative issues, and misinformation about legal aspects were identified. It was noted that one in five immigrants has experienced at least one barrier to accessing the Spanish healthcare system. Barriers to access to the health system are clearly shared by both immigrants and healthcare professionals. Barriers to access to the health system are a result of the coalition of organizational factors, cultural experiences, and socioeconomic and educational determinants. Access to healthcare for irregular migrants in Spain is hindered by language barriers, misinformation, and administrative obstacles, exacerbated by the COVID-19 pandemic. Policies are needed to ensure equitable care, enhance communication, streamline procedures, and strengthen collaboration with non-governmental organizations and cultural mediators to optimize healthcare responses.

## Introduction

In 2020, ∼125 000 irregular immigrants crossed the borders of the European Union, and a large number did so by sea [1]. The definition of an irregular or undocumented immigrant means “a person who, due to an unauthorized entry, non-compliance with an entry condition, or the expiration of the visa, loses a legal status in a transit or host country, including persons who have lawfully entered a transit or host country but overstayed” [2]. Irregular immigration rates have been on the rise since the year 2000 in Spain. In 2023, the nation recorded its second-highest number of irregular migrant arrivals, according to data from the Spanish Ministry of the Interior. By 2024, Spain had become the primary entry point for migrants into Europe [3].

In this country, the legal and administrative framework for irregular migrants is governed by Law 12/2009 of 30 October, which regulates the right to asylum and subsidiary protection for individuals seeking international protection and ensures access to social services for those lacking economic resources, guaranteeing support for individuals unable to meet their basic needs [4]. However, there are still aspects to be designed and consolidated so that irregular immigrants can access national resources and normalize their situation in an operative way. Immigration itself is an important factor in the health of these people, and of the communities that host these groups encounter social and economic challenges that require the openness and reorganization of the national government resources [3]. In this sense, there are few studies that analyze the existing barriers to access to the health system in the immigrant population, and even less if we focus on the public health system of Spain. The last review on this subject [5] highlighted the great difference that existed between immigrants and autochthonous people in terms of accessibility to the health system and clinical interventions for the same health need. It is also striking that there is no recent review of the topic since the beginning of the COVID-19 pandemic until now, even though these barriers, as well as linguistic and cultural barriers, were aggravated by distance and the digital measures initiated in the pandemic [2, 6].

To eliminate existing barriers and ensure adequate healthcare for this population, it is crucial to evaluate the system’s functioning, identify process gaps, and assess health interventions to monitor progress [7]. Further research is needed to gather comprehensive data on aspects such as attendance records, non-governmental organizations (NGOs) contributions, and administrative or insurance information [8]. This highlights the need for a deeper understanding of accessibility within the national health system. For this reason, the objective of this review was to investigate the barriers faced by irregular immigrants upon their recent arrival in Spain when accessing the public health system.

## Methods

### Study design

This study was carried out following the recommendations of the PRISMA methodology [9] (Preferred Reporting Items for Systematic Reviews and Meta-Analyses). During the month of June 2024, a bibliographic search of the available literature was carried out according to the objectives; this research was revised in February 2025. This protocol was registered in PROSPERO with the code CRD42024543990.

The clinical question was posed using the PIE (Patient/Problem/Population; Intervention/Interest; Evaluation) system in order to synthesize the literature review (Supplementary Table 1). The following research question was developed: What barriers and facilitators of access to the public health system do irregular immigrants encounter when they arrive in Spain?

### Databases and search strategy

The search was carried out in the PubMed, CINAHL, Scopus, LILACS, Web of Science, and Enfispo databases. Topic-related MeSH descriptors were identified to define the search equation and combined using parentheses and quotation marks, as well as Boolean operators “AND” and “OR” (Table 1).

**Table 1.** Search strategy and databases

Database	Search strategy	Results
PubMed	(Immigrants OR migrants OR “asylum seekers” OR refugees) AND (healthcare OR “health care utilisation” OR “Health care use” OR “Health care delivery” OR “health services” OR “health services delivery” OR “use of health service” OR “Sociodemographic Determinants” OR “Immigrant assimilation”) AND Spain	852
Scopus	TITLE-ABS-KEY [(immigrants OR migrants OR “asylum seekers” OR refugees) AND (healthcare OR “health care utilisation” OR “Health care use” OR “Health care delivery” OR “health services” OR “health services delivery” OR “use of health service” OR “Sociodemographic Determinants” OR “Immigrant assimilation”) AND Spain]	540
CINAHL	[(Immigrants OR migrants OR “asylum seekers” OR refugees)] AND [(healthcare OR “health care utilisation” OR “Health care use” OR “Health care delivery” OR “health services” OR “health services delivery” OR “use of health service” OR “Sociodemographic”)]	2660
Enfispo	(Immigrants OR migrants OR “asylum seekers” OR refugees) AND (healthcare OR “health care utilisation” OR “Health care use” OR “Health care delivery” OR “health services” OR “health services delivery” OR “use of health service” OR “Sociodemographic Determinants” OR “Immigrant assimilation”) AND Spain	23
LILACS	(Immigrants OR migrants OR “asylum seekers” OR refugees) [Palavras] and (healthcare OR “health care utilisation” OR “Health care use” OR “Health care delivery” OR “health services” OR “health services delivery” OR “use of health service” OR “Sociodemographic Determinants” OR “Immigrant assimilation”) [Palavras] and Spain [Palavras]	5
Web of Science	(Immigrants OR migrants OR “asylum seekers” OR refugees) AND (healthcare OR “health care utilisation” OR “Health care use” OR “Health care delivery” OR “health services” OR “health services delivery” OR “use of health service” OR “Sociodemographic Determinants” OR “Immigrant assimilation”) AND Spain	693

The resulting search strategy was (immigrants OR migrants OR “asylum seekers” OR refugees) AND (healthcare OR “health care utilisation” OR “health care use” OR “health care delivery” OR “health services” OR “health services delivery” OR “use of health service” OR “sociodemographic determinants” OR “immigrant assimilation”) AND Spain. A reverse search to collect suitable research from the reference lists of other shortlisted studies was performed.

### Eligibility criteria

Specific inclusion and exclusion criteria were applied to select articles. Only studies conducted in Spain involving irregular immigrants and healthcare professionals were included, focusing on barriers related to bureaucracy, documentation, direct care, and healthcare accessibility and equity. Reviews, meta-analyses, dissertations, brief reports, conference proceedings, commentaries, and editorial articles were excluded.

### Study risk of bias and methodological assessment of quality

A bias assessment was carried out to know the reliability and relevance of the studies and, therefore, their eligibility. To do this, the critical appraisal tools of the Joanna Briggs Institute (JBI) [10] were used. Two reviewers participated in this process, and a third reviewer acted in case of discrepancy. In this assessment, a cut-off point was established for scores equal to or greater than half of their maximum value according to the number of items; for quantitative research, cross-sectional analytical studies were measured through eight items (Supplementary Table 2), and scores of 4/8 or higher were accepted; for qualitative research, 10 items were evaluated (Supplementary Table 3), and scores of 5/10 or higher were accepted. In this study, the researchers did not exclude any studies, as they all exceeded the minimum required scores, and there were no discrepancies between reviewers.

### Flowchart of the study using the PRISMA methodology

**Figure 1.**
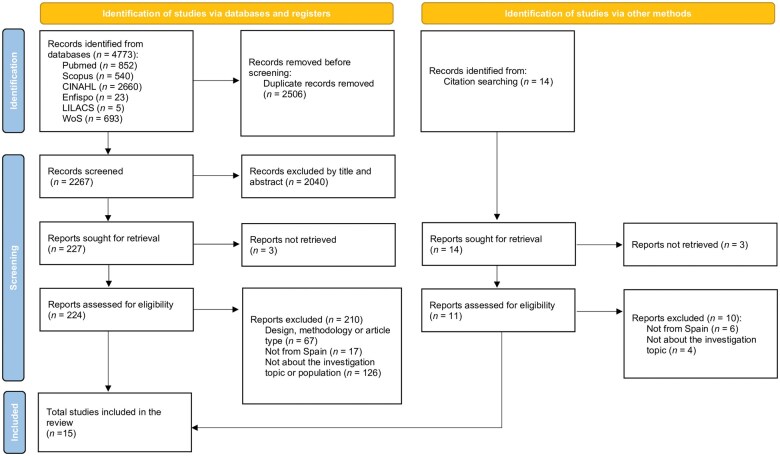
PRISMA flow chart.

The flowchart followed the PRISMA methodology [9], reflecting the results of the search and the reasons for the exclusion of discarded articles (Figure 1). This review began with a total of 4773 studies after conducting database searches, of which *n *= 2267 were screened following the removal of duplicates (*n *= 2506). Of these, *n *= 224 articles were selected for full reading, while *n *= 67 were discarded due to issues related to design, methodology, or article type. Additionally, *n *= 17 articles that did not focus on Spanish territory were excluded, along with *n *= 126 articles that either did not meet the objectives of this research or investigated irregular or undocumented immigrants. One study was added to this selection through a snowball search. Ultimately, 15 studies were selected for this review.

## Results

### Description of the characteristics of the selected studies

Fifteen studies were selected in this systematic review [2, 11–24] (Table 2).

**Table 2.** Summary of selected articles

Author, year of publication, country and reference	Objective of the research	Study design 1. Type of research 2. Type of intervention 3. Main variables of interest	Sample	Main results and conclusions
Jiménez-Lasserrotte *et al*., 2023, Nationwide (Spain) [2].	Describe and understand irregular migrants’ experiences of health disparities while living in squatters during the COVID-19 pandemic.	1. Qualitative study. 2. Seventeen in-depth interviews were conducted by researchers in English, French, and Arabic. Both verbal and non-verbal information are collected. For their analysis, they used the ATLAS computer program. 3. Difficulty in accessing the health system and the therapeutic approach to communication. In addition to being interesting the perspective of this sector of the population in the face of the problems encountered in the vaccination of this disease, it was one of the most debated topics.	There was a total sample of 34 irregular migrants from different African countries living in illegal settlements.	This study was divided into subsections. Difficulty in accessing the health system: One of the main barriers after COVID-19 was the imposition of telephone appointments, this being an exclusion for immigrants due to the language barrier. Another of the barriers expressed was access to health centers or hospitals due to the low economic level they cannot travel by public transport or their own vehicles. Therapeutic approach to communication: They expressed concern about the increased lack of tolerance of professionals in times of COVID. This negative attitude had an impact on the quality of healthcare, affecting access, equity and respect for immigrants. Again, the problem of the language barrier is mentioned, such as a lack of active listening on the part of professionals, but also the lack of respect and empathy on the part of health professionals. The results of this study emphasized the figure of the cultural mediator as a necessity for the migrant population COVID-19 vaccination: One of the major issues that was debated was this due to the disagreement between these patients in their answers. Some of them criticized the lack of information in their own language about this vaccine, having to resort to other alternative sources to obtain this information. Another fear was about the legal rights to access this service.
Ndumbi *et al*., 2018, Nationwide (Spain) [11].	To investigate the progress of immigrants’ access to health services in Europe, focusing on the Spanish results of this study.	1. Descriptive cross-sectional study. 2. Cross-sectional electronic surveys were carried out to investigate barriers to access to the health system. Data are evaluated through the *P* value, the odds ratio, prevalence, and frequencies. 3. Issues such as barriers to access to healthcare among immigrants living with HIV.	There was a total sample of 765 HIV-positive immigrants.	22% of immigrants, i.e. one in five, said that they had experienced at least one barrier to accessing the Spanish health system. The main barriers were long waiting times to get a medical appointment (9%) or at the diagnosis (7%) and lack of health therapy (7%). Being an immigrant was a risk factor for having higher barriers to access for both men (OR: (4.0 [95% CI: 2.2–7.2]) and women (OR: (10.5 [95% CI: 3.1–34.8]).
Bradby *et al*., 2020, Europe, depicted into data from Spain [12].	Analyze how national health systems address the healthcare needs of forced migrants, focusing on the factors that hinder access at the organizational and individual levels.	1. Qualitative study. 2. It was carried out through interviews, which were part of the MigHealthecare project. All interviews were translated into English and then summarized, although they may have lost nuances of the mother tongue. 3. The subject covered by this study is access to health systems, among others. It is only here that Spain is mentioned.	Three focus groups in Spain took part, which included healthcare providers, policy makers and representatives from NGOs.	Barriers to access were the lack of interpreters and cultural mediators in Austria, Greece, Italy, Malta, Spain, Germany, and Cyprus. The lack of linguistic interpretation services prevented verbal communication, which was one of the problems for access. In France, Germany, Italy, Spain, Cyprus, and Greece, it was observed that budget cuts that led to a reduction in staff and equipment had detrimental effects at different levels and times of the health system, being detrimental to all types of patients, more specifically immigrants.
Gimeno-Feliu *et al*., 2021, Aragon, Spain [13].	To understand how immigrants use health services according to their needs by comparing healthcare use between irregular immigrants, documented immigrants, and Spanish nationals in a Spanish region.	1. Retrospective cross-sectional study. 2. Clinical and administrative data from people assigned to all public primary care centers in Aragon, Spain, were analyzed. All statistical analyses were performed using STATA/CI 12. 3. The topics to be highlighted were the condition of access to the health system and use of healthcare.	The sample consisted of 1 070 715 people: 930 131 of Spanish nationality; 123 432 documented migrants; and 17 152 irregular migrants.	After carrying out the study, it was revealed that the use of the public health system by irregular immigrants was lower than that of documented immigrants and Spanish natives, both in terms of the number of visits and hospitalizations per year. Primary care, 0.5% (irregular immigrants), 4.0% (documented immigrants), and 6.7% (natives); with respect to outpatient hospital visits, 0.2 vs. 1.8 vs. 2.9; in terms of planned hospital admissions (per 100 people), 0.3 vs. 2 vs. 4.2; related to unplanned hospital admissions (per 100 people), 0.5 vs. 3.5 vs. 5.2, and emergency room visits (per 10 people), 0.4 vs. 2.8 vs. 2.8. Focusing on pharmacies, spending was lower among irregular immigrants than among documented immigrants and Spanish nationals: €8.7 compared to €77.4 compared to €366.5. The data were related by age and revealed a lower use of health services by irregular immigrants at all levels of care. The decrease in percentage of access to the health system for immigrants may be smaller due to accessibility barriers related to their work circumstances, in addition to feelings such as fear and shame or even discrimination by health workers, which makes immigrants feel rejected. It was all related to a lack of information, language barriers, and financial difficulties. Another potential barrier to access to healthcare is the fear that user data will be transferred to authorities, which could result in deportation from the country.
Gil-Salmerón *et al*., 2021, Europe, depicted into data from Spain [14].	Assess immigrants’ perceived health discrimination, access to health services, and the availability of translation services in health systems.	1. Descriptive cross-sectional study. 2. Descriptive statistics and multivariate regression analyses were performed to investigate risk factors for discrimination. The questionnaires were translated into different languages to make them easier for immigrants to read. They were evaluated through the DMS scale. 3. The main topics of interest were: access to healthcare services and translation and discrimination services in medical settings.	Total sample: 1294 Interviewees in Spain: 202 Selected over 18 years of age, through the snowball method in 10 European countries.	In Spain, only 27.36% needed access to health services and had it. It is a low figure, although compared to the rest of the countries united in this survey it is the second with the best results. On the other hand, the percentage of the question regarding worse access to health services compared to the local population is 19.9%, this being the second lowest figure compared to other countries. In terms of barriers to health access, the country of access and not speaking the language of the host country is associated with greater discrimination. Younger immigrants, women, and chronic immigrant patients experienced greater discrimination. All this has been increased due to the pandemic.
Pérez-Urdiales *et al*. 2019, Basque Country, Spain [15].	To investigate the experiences and feelings of migrant women from sub-Saharan Africa about access to the public health system in the Basque Country.	1. Qualitative methodology. 2. The study consists of an interview, which begins with an open-ended question and is followed by a semi-structured guide. The data were dumped into the qualitative data analysis software *Open Code* 4.03 to have the information obtained in order. The analysis of the study was developed following the approach given by Graneheim and Lundman. 3. The highlighted issues are the factors/barriers that caused immigrants to stop seeking medical care, in addition to some facilitators that these patients represent to reduce these barriers.	The sample consisted of 14 women from 8 countries in sub-Saharan Africa. The initials were recruited in the free clinic itself and after this, social organizations were contacted to capture part of the users of that institution.	The factors that are related to the fear of the need for medical care are being rejected due to disadvantaged social position or skin color and lack of knowledge about the rights and laws of the country and the organization of the health system. These are barriers to meeting legal conditions and facilitating access for migrants. Undocumented patients avoid seeking medical attention because they feared that the system could report their data and lead to their deportation or even that they would be asked for money for these health benefits and when they could not pay for them, they would be returned to their country of origin. In health centers, the administrative staff must provide information about the process of access to the health system, the result of this interview reflects that the information was not complete. Another important barrier was the language, in addition to the lack of involvement of some professionals to understand them. On the other hand, it should be noted that professionals developed skills to be able to communicate with these patients, such as attending with people who could translate for them, although this is a conflict due to data privacy.
Ruiz-Azarola *et al*., 2020, Andalusia, Spain [16].	To find out the opinion of immigrants on access to the health system after the entry into force of Royal Decree-Law 16/2012 and on the effect that economic cuts may have had.	1. Qualitative study. 2. Semi-structured interviews conducted in two phases. A total of 60 interviews were conducted. The content analysis was through the Aday and Andersen model. 3. The topics to be highlighted are the barriers to access to the health system, in addition to studying the facilitators. It is worth highlighting the individual health card as a fundamental element at the entrance to health services. Another issue to highlight is the worsening of the access to the health system after Royal Decree-Law 16/2012 came into force.	The sample consisted of 36 people from Bolivia, Morocco, and Romania.	It is divided into three blocks: Entry to the health system: The barriers to access are administrative procedures and the lack of information about them. One of the facilitators of this problem in the first phase of the study was to have an informal network such as family members or society itself, i.e. NGOs. The second phase shows television and digital media as facilitators of information, with the disappearance of NGOs. Entrance to the health system: Health cards are the fundamental element for access to healthcare. Even with the “Individual Health Card,” there are complications such as the misinformation of health professionals regarding these patients. We find other problems such as the incompatibility of migrants’ working hours with healthcare, the cost of transport, or the fear that is created by not having truthful and complete information. There are known situations of discrimination or denial of healthcare to people without clinical history or who do not contribute. The worsening of the situation, due to the Spanish Law 16/2012, is relevant because of the lack of clarity to interpret it for professionals and due to the misinformation of immigrants in the face of this law. In addition, economic cuts, which imply a decrease in the quality of services, a reduction in health personnel, a reduction in hours of care, an increase in waiting lists in primary care, hospitals and emergencies, and an increase in the payment of medicines for immigrants who do not contribute to Social Security is remarked. Characteristics related to the situation of the people interviewed that condition access to healthcare: After Spanish Law 16/2012 came into force, a crisis was perceived, which created an increase in the number of people in an irregular administrative situation, greater job insecurity, a rise in the price of medicines and a decrease in resources for transports. Barriers to access, such as discrimination, worsened if they were undocumented or unemployed; the difficulties in accessing the health system increase even more due to the higher economic cost and less prevention of diseases.
Hsia and Gil-González, 2021, Nationwide (Spain) [17].	This study aimed to analyze the legislative and administrative challenges in implementing the 2018 Royal Decree-Law, which sought to expand healthcare access for undocumented immigrants in Spain. It also examined these barriers from the perspective of healthcare providers and system administrators.	1. Qualitative study. 2. Twelve in-depth interviews were conducted with participants in Spain. The focus was on gathering information through interviews to understand the participants’ perspectives on access to healthcare for undocumented immigrants in Spain. 3. The study examined various aspects of access to healthcare for undocumented immigrants in Spain, focusing on participants’ experiences and perspectives. Key areas of analysis included participants’ knowledge of the steps required for undocumented immigrants to obtain healthcare, as well as the challenges and barriers they face, such as legislative and administrative obstacles.	The sample for this qualitative study comprised 12 participants from Spain. Specifically, the study included interviews with 7 men and 5 women. The participants consisted of Spanish healthcare workers, government officials, hospital administrators, and individuals affiliated with non-governmental organizations (NGOs).	The 2018 Royal Decree Law imposes a 3-month residency requirement for irregular immigrants to access public healthcare, creating significant barriers due to difficulties in proving residency and inconsistent official interpretations. Additionally, the lack of clarity regarding the applicability of exceptions from the 2012 RDL for vulnerable groups leads to uncertainty and complicates access to care. Spain’s territorial organization into autonomous communities causes jurisdictional confusion, resulting in disparities in healthcare access. Irregular immigrants also face challenges obtaining necessary documentation and experience erosion of trust in the government and healthcare system due to past restrictive policies. On the other hand, NGOs provide vital support to irregular immigrants by facilitating access to services and advocating for inclusive policies. Furthermore, the legacy of a universal healthcare system prior to 2012 continues to influence efforts for equitable access to medical care for all.
Jiménez-Lasserrotte *et al*., 2023, Nationwide, Spain [18]	The primary objective of this study was to describe and understand the experiences of healthcare providers regarding the health needs and emergency care processes for irregular migrant children arriving in Spain by small boats.	1. Qualitative study. 2. Two focus groups (FGs) were conducted, each consisting of five health professionals. The focus groups lasted an average of 62 min. Additionally, eleven in-depth interviews were carried out. 3. The primary variables of interest analyzed in the study included: the health needs of migrant children upon their arrival in Spain; the social care provided to both unaccompanied minors and those traveling with adults; the challenges and progress in addressing the needs of migrant children; and the perceptions and experiences of healthcare providers involved in their care.	The sample of this qualitative study consisted of 21 healthcare providers, including professionals from the health and social sectors, who were part of the emergency team.	The study examines the facilitators and barriers to providing support for migrants. Effective interinstitutional coordination among emergency teams, such as the Red Cross, Maritime Rescue, and child protection services, enhances the efficiency of responses to migrant needs. The involvement of cultural mediators fluent in multiple languages also improves communication and culturally appropriate care. However, several barriers persist. Challenges in information transfer and coordination with police hinder the continuity of medical care. Legal issues arise from the lack of documentation and difficulties in verifying migrants’ ages, which complicate access to necessary services. Additionally, migrant children face specific health problems related to their journeys, compounded by limited access to mental health support. Negative societal attitudes toward migrants further obstruct their integration and access to services, creating ethical dilemmas for healthcare providers. Resource limitations and language barriers additionally restrict the provision of comprehensive care. Lastly, a lack of knowledge regarding migrants’ rights and service access procedures poses significant challenges for this population.
Pérez-Urdiales and Goicolea, 2018, Basque Country, Spain [19].	To investigate the feeling of health professionals working in health centers about the barriers and facilitators in immigrant women’s access to general and sexual and reproductive health in the Basque Country.	1. Qualitative study. 2. Individual interviews. A semi-structured question guide was used for this purpose. All interviews were audio-recorded, transcribed, and imported into Open Code 4.03 software. 3. Four categories stand out that indicate the barriers and facilitators to national and regional health services for immigrant women, based on the characteristics of immigrant women themselves, the attitude of administrative and health personnel, the functioning of the health system, and finally, the policies that regulate access to health services.	The sample consisted of 11 health professionals, 10 women, and 1 man. Of them, six were doctors, four were nurses, and one was a clinical psychologist.	There were differences in immigrant women’s access to health services depending on their origin. It was identified that certain cultural or religious conceptions may have a greater influence on access for Maghrebi women, such as the preference to be attended by a woman or the embarrassment of being explored. African women are the ones who face the most problems in access due to language. Other barriers to health access include having a job, fear of rejection by health system personnel, the social support network and gender-based violence. Another factor that influences is the attention received from the administrative staff. Influencing factors were the predisposition when talking to these patients, empathy, time and changes in administration routines, in addition to being influenced by social prejudices. The treatment of immigrant women by health workers was good compared to that of administrative staff. All participants agreed that the biggest barrier is the Spanish Law 16/2012, due to the ignorance about the right to receive health services and the fear that they will be billed. The fear of being recognized as irregular immigrants by public institutions and its possible legal consequences were pointed out as barriers to access.
García-López *et al*., 2024, Andalusia, Spain [20].	To describe and analyze the experiences of irregular migrant men throughout their migration process and reception in Spain during the COVID-19 pandemic.	1. Qualitative descriptive study. 2. The data were collected between January and March 2023 through 16 in-depth individual interviews. These interviews took place at an office of the Humanitarian Reception Centers, where immigrants resided for several months after their arrival in Spain. The data were analyzed using thematic analysis. 3. Three main themes emerged: (i) The impact of the COVID-19 pandemic in prompting irregular male migrants to leave their countries of origin. (ii) The effects of COVID-19 lockdown policies on the migratory journey. (iii) The challenges of housing and social integration for irregular male migrants during the pandemic, characterized by isolation and loneliness.	The sample of this study comprised 16 irregular African migrant men who arrived on the Spanish coast by boat during the COVID-19 pandemic. The participants had a mean age of 24.2 years.	The study highlights that irregular migrant men faced multiple barriers in accessing Spain’s public healthcare system during the COVID-19 pandemic. These obstacles emerged at various stages of their migratory journey and reception in the host country: Stigmatization and discrimination: Migrants were often perceived as virus spreaders, leading to discriminatory attitudes and xenophobia. Bureaucratic and legal barriers: Their irregular migration status limited access to formal employment and state benefits, increasing their vulnerability to the virus.
Serre-Delcor *et al*., 2021, Nationwide (Spain) [21].	To describe, from the point of view of health professionals, the health needs and barriers faced by newly arrived immigrants to Spain when accessing the health system.	1. A quantitative descriptive cross-sectional study. 2. Self-administered questionnaires consisting of 22 items were carried out. The statistical analysis included measures of distribution, central tendency, and dispersion. The statistical analysis was performed using the SPSS 23.00 program. 3. The topics on which information was obtained were the following: Health status of immigrants. Use of health services by immigrants. Knowledge of immigrants’ health rights. Strategies to improve the use of and accessibility to health facilities for migrants.	There were 228 health professionals who completed the survey.	The health status of the native population is much better than that of the immigrant population, with those from sub-Saharan Africa standing out as the worst health. Mental health disorders suppose the highest percentage of illnesses in immigrants. About the use of health services, 117 out of 217 (53.9%) professionals indicated that the native population abused health services. However, only 26 out of 224 (11.6%) professionals believed that immigrants overuse the health system. The study found that only 28.9% of participants had acquired knowledge about migrants’ health rights through specific training provided in their workplace. There are four barriers to access to healthcare: language, cultural differences, administrative issues and fear of being undocumented. The strategies given by healthcare professionals to improve access for immigrants were greater training in intercultural skills, greater availability of community health agents and translators, and greater efforts to train migrants in the use of the health system.
Pérez-Urdiales, 2021, Basque Country, Spain [22].	This study aimed to analyze access to public health services for immigrant women and undocumented immigrants in the Basque Country. It focused on evaluating the effects of restrictive conditions on primary health service access, understanding healthcare professionals’ perceptions of barriers and facilitators, and exploring the experiences of sub-Saharan immigrant women regarding appropriate health services.	1. Mixed methods study. 2. A quantitative study was conducted using a segmented time series design to compare trends in the number of undocumented immigrant consultations at a free primary care clinic before (2007–2012) and after (2013–2017) the implementation of the 2012 decree. Additionally, two qualitative studies were carried out through interviews to explore healthcare professionals’ perceptions and the experiences of immigrant women regarding access to health services. 3. This study examines factors affecting immigrants’ access to healthcare services, focusing on the number of primary care consultations by undocumented immigrants, healthcare professionals’ perceptions of barriers and facilitators, and the experiences of Sub-Saharan immigrant women in the Basque Country. It also considers the legal and political context and individual characteristics, including ethnicity, gender, migration status, language, socioeconomic status, and exposure to gender-based violence.	The study employed a mixed-methods design, incorporating quantitative and qualitative data. Quantitative Analysis: It analyzed 9272 health consultations for undocumented immigrants from January 2007 to June 2017, with 76.94% involving men and 23.06% women. Qualitative Analysis: It included 11 interviews with healthcare professionals and 14 interviews with female immigrants from eight sub-Saharan African countries who used public health services in the Basque Country.	The primary barrier to healthcare access for immigrants is the lack of legal entitlement, particularly affecting undocumented individuals due to restrictive laws. Additionally, many immigrants are unaware of their rights and face challenges in meeting legal requirements like municipal registration. Access barriers include insufficient information on healthcare services, communication difficulties with staff, and fear of rejection or costs, which deter them from seeking help. Language barriers and stigma related to their immigration status, along with a lack of cultural competence among healthcare providers, further complicate access. Facilitators for accessing healthcare include being informed about rights and services, having support from individuals who can assist in navigating the system, and receiving help from professionals sensitive to their vulnerabilities. Community support from social organizations and free clinics is vital for promoting health access, while cultural mediators enhance communication and understanding, making healthcare more accessible for vulnerable populations.
Plaza del Pino *et al*., 2024, Andalusia, Spain [23].	The aim of this study was to understand the perceptions of undocumented Moroccan immigrants living in shantytowns regarding the healthcare system in southern Spain.	1. Qualitative study. 2. Individual interviews. A semi-structured question guide was used for this purpose. 3. The research examined several key factors affecting immigrant health. It focused on the impact of slum living conditions on health, access to and utilization of healthcare services, and the quality of care received by immigrants.	The qualitative study sample consisted of 24 undocumented Moroccan immigrants, comprising 11 men and 13 women.	The primary barrier was communication issues, particularly language difficulties, which hindered immigrants’ rights to receive health information. Discriminatory attitudes and a lack of cultural competence among healthcare staff also contributed to negative experiences. The shantytowns’ remote locations limited access to healthcare services, while ongoing economic hardships and social challenges, such as sexual harassment, exacerbated stress. Despite having health cards, many immigrants felt neglected by the healthcare system and perceived the quality of care as inadequate, with little follow-up or proper diagnostics. On the positive side, all participants had temporary health cards, allowing access to public healthcare, often facilitated by NGOs. Informal translation methods, including non-verbal communication and assistance from acquaintances, helped overcome language barriers. Participants who spoke Spanish were better able to navigate healthcare services. They also suggested that having intercultural mediators within the healthcare system would improve communication and understanding.
Sahraoui, 2024, Ceuta and Melilla, Spain [24].	The primary aim of this study is to examine the implications of Ceuta and Melilla as fronts of “Fortress Europe” within the framework of entrenched postcolonial hierarchies.	1. Qualitative study. 2. Interviews were conducted with healthcare professionals, migrant women, and social workers to gather in-depth insights into their experiences and viewpoints. In Melilla, 24 individual interviews and 6 focus groups were held with healthcare providers. Additionally, 18 interviews were conducted with women living in the Temporary Immigrant Accommodation Center, along with discussions with most of the social workers active at the center at that time. 3. The primary areas of interest include the impact of immigration status on access to rights, EU migration control policies in Ceuta and Melilla, and the establishment and maintenance of borders. The study also addresses access to basic social services, narratives of multiculturalism and exclusion, and the colonial history of Ceuta and Melilla, which shapes current social and political relations. Additionally, it examines the role of social actors in these contexts.	A total of 24 individual interviews and 6 focus groups were conducted with healthcare professionals working at public hospitals and NGOs in Melilla. Additionally, 18 interviews were held with women residing in the “Temporary Immigrant Reception Center.”	The study highlights several significant barriers to accessing public healthcare in Ceuta and Melilla for immigrants. Policies of “internal externalization” and deterrence have transformed these cities into frontiers of “Fortress Europe,” limiting access to basic services, including healthcare. Immigrants, particularly undocumented Moroccan residents, face specific restrictions such as the requirement of a visa for municipal registration, a critical step for regularization. Administrative and economic barriers also exist, with pregnant women facing additional obstacles like needing proof of residence, verified by police, to access maternal health services. Moreover, healthcare is often used as a tool for migration control, with the payment of hospital bills linked to regularization, disproportionately affecting women. The lack of recognition of cultural diversity, combined with police involvement in healthcare procedures, exacerbates exclusion, contributing to a vicious cycle of limited access to services. On the other hand, the study identifies certain facilitators within this context. Despite the challenges, various social actors, including healthcare providers and NGOs, play a crucial role in assisting immigrants. However, the study also notes the potential for further improvement in facilitating access to healthcare services, especially if multicultural policies and recognition of linguistic diversity, such as the use of Darija or Tamazight, were implemented. The cooperation between social actors and the use of technology, such as facial recognition to ensure payment, also contributes to shaping the immigrant experience. Although these elements can help improve access, the overall barriers still present significant challenges to achieving comprehensive healthcare access for undocumented migrants in these regions.

Summing up samples, this review consisted of a total of 28 946 people. The article with the largest sample was the one carried out by Gimeno-Feliu *et al.* [13] with 140 584 participants from the EpiChron Cohort, and the study with the smallest sample was the one carried out by Pérez-Urdiales *et al.* [19] with 11 participants. Among the informants, 10 studies included immigrants as participants [2, 11, 13–16, 20, 22–24], while 7 studies also gathered information from healthcare professionals [12, 17–19, 21, 22, 24].

Ten articles used a qualitative methodology [2, 12, 15–20, 23, 24]; only one was conducted from a mixed perspective [22] and another four articles were conducted through a quantitative methodology [11, 13, 14, 21]. The data were collected in 2007–2017 [22], 2011 [13], 2009–2010 and 2012–2013 [16], 2013–2015 [11], 2015 [19], 2016–2017 [15, 24], 2017–2018 [12], 2018 [21], 2018–2019 [14], 2019–2020 [17], 2022 [2, 23], 2022–2023 [18], and 2023 [20]. These studies were carried out in Spain [2, 11, 13, 15–24], although two articles were developed in Europe with disaggregated data on Spain [12, 14]. Studies were found from different regions of Spain: Andalusia [16, 20, 23], Ceuta and Melilla [24], the Basque Country [15, 19, 22], and Aragon [13]; the rest of the articles were carried out at the national level [2, 11, 12, 14, 17, 18, 21].

Most of the included studies were qualitative. The JBI scores for these studies ranged from 7/10 to 10/10, indicating good to excellent overall quality. Quantitative studies also received relatively acceptable JBI scores, ranging from 4/8 to 8/8, suggesting that these designs may also be appropriate. A mixed-methods study demonstrated a high level of methodological rigor, achieving the maximum JBI scores for both qualitative (10/10) and quantitative (8/8) components.

### Barriers to accessing the healthcare system for the immigrant population in Spain

#### Healthcare system utilization

After the arrival of irregular immigrants in Spain, the most reported diseases are mental health disorders, infectious diseases, and other non-communicable systemic diseases [21]. Several studies showed that a high percentage of immigrants had never gone to a health center since they arrived in Spain, resulting in the number of irregular immigrants accessing health services being lower than the number of documented people and natives [14, 22]. Gimeno-Feliu *et al*. [13] studied the use of the public health system by irregular migrants in 2011, considering both the number of visits and hospitalizations per year. In primary care centers, an average of 0.5 (SD = 2.0) visits per year by irregular migrants was recorded, in contrast to 4.0–6.7 visits per year by regular migrants and Spanish natives, respectively. Regarding home care, irregular migrants received only 0.2 (SD = 1.3) visits per year, compared to 1.8 and 2.9 visits for documented migrants and natives. Concerning hospital admissions, 0.3 (SD = 5.4) and 0.5 (SD = 8.1) irregular migrants per 100 individuals were admitted, both planned and unplanned, compared to 4.2 and 5.2 native individuals in each type of admission. Similarly, emergency care admissions per 10 individuals were 0.4 (SD = 3.2) for irregular migrants, compared to 2.8 admissions for documented migrants and Spanish natives [13].

Women, followed by young people and patients with chronic diseases, face the most discrimination in the health system [11, 14]. There is evidence that, in general, women encounter greater barriers to accessing the health system than the rest of the groups [11]. In addition to this, cultural differences are relevant depending on where immigrants come from. For example, women who came from Algeria, Morocco, and Tunisia reflected greater conflicts when accessing the health system due to their preference to be treated by women, problematic work schedules, the fear of rejection by professionals, or cases of gender violence in the workplace [19].

#### Communication barriers

With respect to the language barrier, several articles mentioned communication problems faced by immigrants [12, 14, 15, 18, 22]. The lack of knowledge of languages on the part of migrants and professionals [19, 21] is one of the main reasons that can discriminate against access to the system and lead to difficulties [14], such as the fear of this population of not obtaining truthful and complete information [16], problems in obtaining an appointment with a health professional, or possibly misguided diagnoses and treatments [15]. The lack of linguistic interpreters and cultural mediators is also a barrier to verbal communication [12], and greater complexity in terms of language has been recognized when patients are African women [19].

#### Lack of information

In reference to misinformation about access to the health system, four articles have reflected the deficit of truthful and adapted information on legislative aspects and the register in the National Individual Health Card [15–17, 23]. A main barrier for immigrants is the great difficulty in complying with certain legal requirements to obtain complete healthcare, such as being linked to the country’s coverage and contribution system or being registered and domiciled in the same Autonomous Community for at least three consecutive months [16, 17]. Misinformation about formal pathways to the healthcare system can lead to compounded costs, as well as a decrease in potential healthcare resources and delays in seeking healthcare [16].

Likewise, both immigrants [19] and health system administrators [15] had low knowledge of legal requirements, leading to problems in interpreting and applying regulations. This lack of knowledge about the handling of their personal and clinical data means that many people do not look for assistance due to the fear that their personal data will be reported to their countries of origin, since they think that they could be deported or that they will be asked for money for health costs [15, 19] and, being unable to pay, they will still be deported [15]. Thus, in relation to communication problems and misinformation about the mechanisms of the system, there are great gaps of information in their medical histories, including interventions or assistance received, both in their country of origin and upon arrival in Spain [2, 15, 18, 23].

#### Undocumented status and irregular immigration situation

On the other hand, it has been possible to identify a series of barriers that occur due to the employment and economic situation of immigrants. Healthcare is affected by immigrants’ lack of time, which is often related to their working schedules, irregular contracts, lack of occupational insurance, and fear of losing their jobs [16, 19]. It is also mentioned that the most common transport among immigrants is on foot, on scooters, or bicycles, so it is often necessary to use others’ vehicles or public transport to attend a health center, something that is not always within their economic range [16]. Another of the fears is having to pay for the services received, influenced by the lack of knowledge [15]. The lack of knowledge about the system characteristics causes that not all immigrants benefits from it, harming their health [13, 18].

### Facilitators of access to the health system for the immigrant population in Spain

Regarding the facilitators for the language barrier, Pérez-Urdiales *et al*. [15] expressed that immigrants themselves are aware of the capacities that professionals have to be able to communicate with them. They also emphasize the communication facilities that can be made, thanks to the assistance of a translator [15, 23], although this is considered a conflict for data protection and privacy. On the other hand, Arab women mentioned that having contact with a cultural mediator in hospitals and health centers helps reduce the impact of the communication barrier [2, 19, 24]. Other factors that have been highlighted in this review are the presence informal companion, such as family, friends, digital social networks, or communities formed in NGOs [16, 19].

Finally, health centers must be useful to improve the health of all users and help access to the system [19], and it has been proposed that undocumented immigrants should be able to pay for the necessary medicines according to their financial capacity and that they should have universal coverage and access to healthcare [2].

### Perspectives of healthcare professionals

The healthcare professionals interviewed in the studies emphasize the multiple barriers that immigrants face in accessing the public healthcare system, including linguistic, cultural, administrative, legal, economic, and attitudinal barriers. At the same time, they identify the specific health needs of immigrants and the negative impact of restrictive immigration policies on their access to medical care.

#### Barriers to accessing the healthcare system

Healthcare professionals identify several barriers that hinder immigrants’ access to medical services, including linguistic and cultural barriers, communication difficulties due to language differences, and cultural misunderstandings [18, 21, 22]. The absence of interpreters and cultural mediators exacerbates the problem. Administrative and legal obstacles also play a crucial role, with professionals describing the difficulties immigrants face due to a lack of documentation and awareness of their rights [12]. Restrictive legislation, such as Law 16/2012, creates uncertainty and fear among immigrants, discouraging them from seeking medical assistance [19, 21, 24], while the fear of deportation prevents many undocumented immigrants from accessing healthcare services [18, 19, 21, 24].

Regarding the health needs of immigrants, professionals point out that mental health issues are prevalent among this population due to migration-related stress, discrimination, and difficulties in integration [18, 21]. Poor living conditions, a lack of preventive care, and traumatic migration experiences contribute to specific health problems, particularly among migrant children [18], who often lack access to mental health support. Some healthcare providers’ biases and prejudices worsen care quality, leading to treatment disparities [17, 18]. The limitations of the healthcare system, including budget cuts, staff shortages, and inadequate training in intercultural competence [12, 19], restrict access to appropriate care. Additionally, the decentralized Spanish healthcare system leads to regional disparities in access, with varying administrative requirements and interpretations of healthcare laws across regions [17, 19, 21].

#### Facilitators of access to the health system

Healthcare professionals emphasize the importance of training in intercultural skills and immigrant rights to improve the quality of care. Cultural mediation and interpretation are essential for enhancing communication and ensuring culturally competent care [22]. The presence of interpreters and cultural mediators is appreciated to reduce language and cultural gaps, improving healthcare outcomes [12, 18, 21, 24]. Similarly, effective interinstitutional coordination strengthens the healthcare response to immigrant needs [18]. NGOs equally perform a fundamental task by providing information, support, and advocacy, helping immigrants navigate administrative and legal barriers. Some professionals develop alternative communication strategies, such as seeking informal interpreters or using non-verbal communication techniques [24].

## Discussion

The primary aim of this research was to examine the barriers faced by irregular immigrants in accessing the public health system in Spain. To summarize the key findings of the study, Table 3 provides an overview of the barriers and facilitators to access, from the perspectives of healthcare professionals and the immigrant population.

**Table 3.** Compilation of key results

Category	Immigrants reports	Healthcare professionals reports
Barriers	Communication problems due to lack of language knowledge. Misinformation about legal aspects and requirements to access the healthcare system. Fear of deportation and legal repercussions. Economic and labor difficulties.	Linguistic and cultural barriers that compromise the quality of care. Administrative and legal obstacles, such as lack of documentation. Prejudices and biases of some healthcare providers. Limitations of the healthcare system, such as budget cuts and lack of training in intercultural competence.
Facilitators	Use of translators and cultural mediators. Support from NGOs and digital social networks. Informal accompaniment by family and friends.	Training in intercultural skills and immigrant rights. Effective inter-institutional coordination. Alternative communication strategies, such as the use of informal interpreters.

One in five immigrants has experienced at least one barrier to accessing the Spanish healthcare system since their arrival in the country [11]. These barriers are not limited to the problems that may occur during initial emergency care derived from the migratory journey, but, sometimes, the presence of barriers continues during healthcare for acute and chronic pathologies or long-term recovery and rehabilitation processes [25, 26]. In the wake of the COVID-19 pandemic, gaps in the healthcare system were exacerbated by misinformation, legislative and administrative barriers, and digital exclusion [2, 14]. Cultural differences were accentuated, and the saturation of health centers and the decrease in face-to-face appointments, which were changed to telephone appointments, worsened the language barrier [2, 6, 14]. Indeed, despite Spain’s legislation granting healthcare access to all residents, including undocumented migrants, challenges and ambiguities in legal entitlements, conflicts between national and regional authorities [17], and difficulties in obtaining necessary documentation for healthcare access emerged during the pandemic [27]. Similarly, the rapid digitalization of healthcare services during the pandemic disproportionately affected these vulnerable populations, who often lacked eHealth literacy and access to necessary technology. Immigrants referred to the telematic systems as unfair [28]. In this sense, the migrants criticized the lack of culturally adapted information about the disease and the legal rights to access vaccination, although NGOs cooperation networks facilitated this labor [2]. Collateral effects were also perceived by migrant workers, whose precarious working and living conditions made adherence to COVID-19 prevention measures challenging. In fact, job loss, precarious living conditions, housing insecurity, irregular administrative status, and limited access to the healthcare system may have been main reasons why the immigrant population showed exacerbated levels of psychological stress during the pandemic [2, 29–31]. The lessons learned from the pandemic imply the necessity of resilient, interoperable, and community-centered health infrastructures that can adapt to emerging challenges [32]. By integrating diverse population data, policymakers can design more equitable health strategies, while healthcare providers can tailor interventions to better address the specific needs of culturally diverse communities [33].

The facilitators expressed in this review were focused on the improvement of communication, i.e. translators or family members who understand both languages or cultural mediators in hospitals and health centers, making community health agents visible, and the help of NGOs and associations [34]. In this sense, community services must be integrated with the existing infrastructure of the health system and are ought to be aligned with the plans and protocols of national and regional authorities [35]. Examining various EU countries that have implemented initiatives to improve healthcare access for irregular migrants, several successful interventions in Germany, Norway, Italy, and Belgium merit attention [36]. For instance, some cities have established municipal health teams or medical centers dedicated to providing treatments that irregular migrants cannot access through national healthcare systems. These services often operate in collaboration with NGOs and aim to offer comprehensive care, including preventive and integration services. Additionally, these centers provide support through interpreters and cultural mediators who assist patients in navigating legal and social challenges. Nevertheless, policy harmonization remains an issue to be addressed. Aligning national policies with international standards and encouraging local governments to leverage community resources are essential steps toward reducing healthcare disparities [37]. This constitutes a shared responsibility among all stakeholders involved in the migration process, whether in healthcare, administration, economics, or politics [38, 39].

Among the limitations of this study, there was a low prevalence of studies focused on the barriers to health access according to the vision of irregular immigrants. Another limitation found at the time of the review was that the designs of the studies were mostly qualitative, so the testimonies addressed populations of different origins and contexts of the national territory, with the variability that this entails.

This systematic review highlights the significant barriers irregular immigrants face in accessing the Spanish healthcare system. With the growing number of irregular immigrants, there is a pressing need for an inclusive healthcare approach. Key obstacles, such as language barriers and misinformation, have been worsened by the COVID-19 pandemic, exacerbating healthcare disparities. These issues conflict with public health and human rights principles, requiring urgent policy reforms. Solutions include better access to translators, clearer communication of healthcare rights, and enhanced collaboration between institutions and NGOs to create a more equitable system. Ongoing research and policy efforts are essential to address these systemic challenges and ensure universal healthcare coverage.

## Supplementary Material

ckaf042_Supplementary_Data

## Data Availability

All data are available within this article. The protocol followed is listed in the International Prospective Register of Systematic Reviews (PROSPERO) with code CRD42024543990.
