# Supplementary material for: Barriers to healthcare access for irregular immigrants after their arrival in Spain: a systematic review
Source: Eur J Public Health. 2025 Apr 9;35(3):407–22. doi: 10.1093/eurpub/ckaf042 (PMC12192432; doi:10.1093/eurpub/ckaf042)
Supplement: ckaf042_Supplementary_Data [file ckaf042_supplementary_data.zip › ckaf042_Supplementary_Data/ejph-2024-09-om-0616-File006.docx]

Supplementary Table 1. PIE Question.

| P (Population) | Immigrants arriving in Spain |
| --- | --- |
| I (Interest) | Access to the Spanish public healthcare system. |
| E (Evaluation) | Barriers and facilitators to receiving health care. |
