# Supplementary material for: Barriers to healthcare access for irregular immigrants after their arrival in Spain: a systematic review
Source: Eur J Public Health. 2025 Apr 9;35(3):407–22. doi: 10.1093/eurpub/ckaf042 (PMC12192432; doi:10.1093/eurpub/ckaf042)
Supplement: ckaf042_Supplementary_Data [file ckaf042_supplementary_data.zip › ckaf042_Supplementary_Data/ejph-2024-09-om-0616-File007.docx]

Supplementary Table 2. Critical appraisal using JBI for cross-sectional studies

| Studies | JBI | Inclusion criteria are clearly defined | The participants and the environment are described in detail | Exposure was validly and reliably measured | The criterion used to measure the condition was objective | Confounding factors were identified | Strategies for dealing with confounding factors | Results measured in a valid and reliable way | Appropriate statistical analysis was used |
| --- | --- | --- | --- | --- | --- | --- | --- | --- | --- |
| Ndumbi P, et al. (2018) [11] | 8/8 | Y | Y | Y | Y | Y | Y | Y | Y |
| Gimeno-Feliu LA, et al. (2021) [13] | 5/8 | N | Y | C | Y | Y | N | Y | Y |
| Gil-Salmerón A, et al. (2021) [14] | 8/8 | Y | Y | Y | Y | Y | Y | Y | Y |
| Serre-Delcor N, et al (2021) [21] | 4/8 | N | Y | Y | C | N | N | Y | Y |
| Pérez-Urdiales (2021) [22] | 8/8 | Y | Y | Y | Y | Y | Y | Y | Y |

**LEGEND:**

YES: **Y**  NO: **N** CONFUSING: **C** NOT APPLICABLE: **NA**
