# Supplementary material for: Barriers to healthcare access for irregular immigrants after their arrival in Spain: a systematic review
Source: Eur J Public Health. 2025 Apr 9;35(3):407–22. doi: 10.1093/eurpub/ckaf042 (PMC12192432; doi:10.1093/eurpub/ckaf042)
Supplement: ckaf042_Supplementary_Data [file ckaf042_supplementary_data.zip › ckaf042_Supplementary_Data/ejph-2024-09-om-0616-File008.docx]

Supplementary Table 3. Critical appraisal using JBI for qualitative research.

| Studies | JBI | Is there congruity between the stated philosophical perspective and the research methodology? | Is there congruity between the research methodology and the research question or objectives? | Is there congruity between the research methodology and the methods used to collect data? | Is there congruity between the research methodology and the representation and analysis of data? | Is there congruity between the research methodology and the interpretation of results? | Is there a statement locating the researcher culturally or theoretically? | Is the influence of the researcher on the research, and vice- versa, addressed? | Are participants, and their voices, adequately represented? | Is the research ethical according to current criteria or, for recent studies, and is there evidence of ethical approval by an appropriate body? | Do the conclusions drawn in the research report flow from the analysis, or interpretation, of the data? |
| --- | --- | --- | --- | --- | --- | --- | --- | --- | --- | --- | --- |
| Jiménez-Lasserrotte, et al. (2023) [2] | 8/10 | Y | Y | Y | Y | Y | N | N | Y | Y | Y |
| Bradby, et al. (2020) [12] | 7/10 | Y | Y | Y | C | Y | N | N | Y | Y | Y |
| Pérez-Urdiales, et al. (2019) [15] | 10/10 | Y | Y | Y | Y | Y | Y | Y | Y | Y | Y |
| Ruiz-Azarola, et al. (2020) [16] | 7/10 | Y | Y | Y | Y | Y | N | N | Y | C | Y |
| Hsia & Gil-González. (2021) [17] | 10/10 | Y | Y | Y | Y | Y | Y | Y | Y | Y | Y |
| Jiménez-Lasserrotte, et al. (2023) [18] | 10/10 | Y | Y | Y | Y | Y | Y | Y | Y | Y | Y |
| Pérez-Urdiales & Goicolea (2018) [19] | 7/10 | Y | Y | Y | Y | Y | N | N | Y | C | Y |
| García-López, et al. (2024) [20] | 10/10 | Y | Y | Y | Y | Y | Y | Y | Y | Y | Y |
| Pérez-Urdiales (2021) [22] | 10/10 | Y | Y | Y | Y | Y | Y | Y | Y | Y | Y |
| Plaza del Pino, et al. (2024) [23] | 8/10 | Y | Y | Y | Y | Y | N | N | Y | Y | Y |
| Sahraoui, (2024) [24] | 9/10 | Y | Y | Y | Y | Y | Y | N | Y | Y | Y |

**LEGEND:**

YES: **Y**  NO: **N** CONFUSING: **C** NOT APPLICABLE: **NA**
